# Vaccination against respiratory tract pathogens in primary immune deficiency patients receiving immunoglobulin replacement therapy

**DOI:** 10.5578/tt.202401813

**Published:** 2024-03-26

**Authors:** Makbule Seda BAYRAK DURMAZ, Reyhan YILDIZ, Göksal KESKİN, Seda ALTINER

**Affiliations:** 1 Division of Immunology and Allergy, Department of Internal Medicine, Ankara University Faculty of Medicine, Ankara, Türkiye

## Abstract

**ABSTRACT**

**
Vaccination against respiratory tract pathogens in primary immune deficiency patients receiving immunoglobulin replacement therapy
**

**Introduction:** *Inborn errors of immunity (IEI) increase morbidity and mortal- ity risks, particularly from respiratory tract infections. Hence, vaccination becomes pivotal for IEI patients. This study aims to examine the vaccination and respiratory tract infection rates in a diverse IEI patient cohort undergoing immunoglobulin replacement therapy (IGRT).
*

**Materials and Methods:** *We retrospectively evaluated IEI patients on IGRT at a tertiary care center. Data on vaccinations and respiratory infections were extracted from medical records.
*

**Results:** *The study included 33 patients (mean age= 37.7 ± 11.4 years; 17 male). The most common clinical phenotype in our cohort was primary anti- body deficiencies (90.9%). Only two patients had a genetic diagnosis, both of whom were brothers diagnosed with Wiskott-Aldrich syndrome (WAS). Almost half (48.5%) of our patients had bronchiectasis and 81.8% were on prophylactic antibiotics. All patients with IEI included in the study were regu- larly receiving IGRT. The vaccination rate of patients against respiratory tract infections was 42.4%, 57.6%, and 78.8% for influenza, pneumococcus, and COVID-19, respectively. Only one patient (7.1%) who received the influenza vaccine developed an upper respiratory tract infection. However, viral panel analysis could not be performed for this patient as they did not present to the hospital. The COVID-19 vaccination rate was notably higher than that of other vaccines, likely due to increased awareness during the pandemic, aided by public advisories and media influence.
*

**Conclusion:** *We observed higher vaccination rates for the COVID-19 vaccine compared to other vaccines (influenza and pneumococcal vaccines). Although we observed the potential impact of social and governmental influ- ence in increasing vaccination rates, it is crucial to acknowledge that vaccina- tion decisions in IEI patients must be individualized.
*

**Key words:** *Inborn errors of immunity; primary immunodeficiency disorders; COVID-19; influenza; pneumococcus
*

**ÖZ**

**
Primer bağışıklık yetmezliği hastalarında solunum yolu patojenlerine karşı aşılama
**

**Giriş:** *Doğuştan gelen bağışıklık hatası (IEI), ağırlıklı olarak solunum yolu ile ilgili enfeksiyonlar açısından morbidite ve mortalite risk- lerini arttırır. Bu nedenle aşılama, IEI hastaları için çok önemlidir. Bu çalışma, immünoglobulin replasman tedavisi (IGRT) alan IEI hasta kohortunda aşılama ve solunum yolu enfeksiyonu oranlarını incelemeyi amaçlamaktadır.
*

**Materyal ve Metod:** *Üçüncü basamak bir sağlık merkezinde IGRT kullanan IEI hastalarını retrospektif olarak değerlendirdik. Aşılar ve solunum yolu enfeksiyonlarına ilişkin veriler tıbbi kayıtlardan elde edildi.
*

**Bulgular:** *Çalışmaya 33 hasta (ortalama yaş= 37,7 ± 11,4 yıl; 17’si erkek) dahil edildi. Kohortumuzda en sık görülen klinik fenotip primer antikor eksiklikleriydi (%90,9). Sadece iki hastada genetik tanı vardı ve bu hastalar Wiskott-Aldrich sendromu (WAS) tanısı alan kardeşlerdi. Hastalarımızın neredeyse yarısında (%48,5) bronşektazi mevcuttu ve %81,8’i profilaktik antibiyotik kullanıyordu. Çalışmaya dahil edilen IEI’li hastaların tamamı düzenli olarak IGRT alıyordu. Hastaların solunum yolu enfeksiyonlarına karşı aşılanma oranları sırasıyla influenza, pnömokok ve COVID-19 için %42,4, %57,6 ve %78,8 idi. İnfluenza aşısı yapılan hastaların sadece birin- de (%7,1) üst solunum yolu enfeksiyonu görüldü ancak hastaneye başvurmadığı için viral panel çalışılamadı. Pandemi sırasındaki farkındalık, kamuoyunun tavsiyeleri ve medyanın da yardımıyla, COVID-19 aşılanma oranı (%78,8) diğer aşılara kıyasla oldukça yüksekti.
*

**Sonuç:** *COVID-19 aşısının diğer aşılara (grip ve pnömokok aşısı) kıyasla daha yüksek oranda uygulandığını bulduk. Aşılama oranları- nın artmasında toplumun ve hükümetin potansiyel etkisini gözlemlemiş olsak da IEI hastalarında aşılama kararlarının bireyselleştiril- mesi gerektiğini kabul etmek çok önemlidir.
*

**Anahtar kelimeler:** *Doğuştan gelen bağışıklık hataları; primer immün yetmezlikler; COVID-19; influenza; pnömokok
*

## INTRODUCTION

Inborn errors of immunity (IEI), previously referred to as primary immunodeficiency, encompass a group of genetic disorders that affect various components of the innate and adaptive immune systems, rendering individuals more susceptible to infections. These infections pose an increased risk of morbidity and mortality (1). Consequently, implementing preventive measures against infectious diseases is crucial for these patients. These preventive measures include, but are not limited to, immunoglobulin replacement therapy (IGRT), primarily aimed at protecting against respiratory tract infections (RTIs), tailored vaccination strategies based on the affected immune system component, nutritional support, respiratory exercises, adherence to environmental and personal hygiene guidelines, and antimicrobial drug prophylaxis.

Vaccination is particularly crucial for IEI patients due to their increased susceptibility to vaccine-preventable diseases, but vaccination strategies should be individualized based on the affected immune system component (2). Vaccines are divided into four groups: live vaccines, inactivated vaccines, mRNA and DNA vaccines, and vector-based vaccines. Live vaccines can potentially cause mild or life-threatening diseases in some IEI patients, therefore, the choice for vaccination should be carefully evaluated based on the clinical phenotype and the affected immune system component, with maximum awareness of contraindications (2-4). Inactivated vaccines are

generally safe for individuals with immunodeficiency. However, the effectiveness of these vaccines may vary depending on the level of immunodeficiency. Additionally, immunocompromised individuals are not included in the efficacy and safety studies of vaccines. Data on vaccine response in IEI patients is limited, and only a few reports have been published regarding vaccination (2-6).

The International Union of Immunological Societies (IUIS) IEI Committee has classified IEI into ten classes based on phenotypes in its latest update (7). The most common symptomatic clinical phenotype in the adult IEI patient group is the common variable immunodeficiency (CVID), which is included in the subgroup of primary antibody defects. The primary clinical features of CVID are recurrent RTIs, and the implementation of IGRT reduces the incidence of infections. However, some CVID patients may continue to experience RTIs leading to permanent lung damage (8). To enhance the diversity of immunoglobulins (Igs), treatment products are sourced from over 1000 healthy donors. IGRT aims to supplement the deficient IgG antibodies in patients. However, the antibody concentrations in IgG preparations can vary, and in some cases, they may be insufficient, particularly when dealing with diseases that have low vaccination coverage or are not prevalent in the general population. Furthermore, current IgG preparations may not include antibodies against the most recent strains of the influenza virus

(9). There are also limited studies on vaccine responses

in patients receiving IGRT, and they suggest that some CVID patients may exhibit positive responses to specific vaccines, particularly conjugate vaccines, while others may demonstrate reduced but still protective responses to polysaccharide vaccines (3,10-12).

As a result, some antibody titers may be low in IgG products and IGRT may not provide sufficient amounts of specific antibodies. Therefore, for patients with immune system disorders, inactivated vaccines against respiratory infections such as influenza vaccine, pneumococcal vaccine, and COVID-19 vaccine are recommended, depending on the specific type of immune deficiency and the age of the patient (2-5,13). We aimed to investigate vaccination rates and the incidence of RTI in our IEI patients, focusing on infectious agents such as influenza, pneumococcus, and COVID-19, and to compare the rates of illness and hospitalization due to relevant pathogens between vaccinated and unvaccinated groups.

### MATERIALS and METHODS

**Study Population**

Our retrospective study included patients aged 18 and over who received IGRT IEI and were followed up in our clinic between 2019 and 2022, with accessible medical records. The patients were diagnosed according to the criteria of the European Society for Immunodeficiencies (ESID) and phenotypically classified according to the IUIS (7,14). Demographic characteristics and laboratory and imaging results of the patients were obtained from electronic records. Vaccination rates of the patients against influenza, pneumococcus, and COVID-19, which are RTI agents, and their post-vaccination infection status with the relevant agents, and the infection status of the unvaccinated patient group with the relevant agents were recorded. Infection status and hospitalization rates of vaccinated and unvaccinated patient groups were compared. Patients whose full information could not be obtained from the electronic records were excluded. The study protocol was approved by our University’s Ethics Committee (approval number: İ04-229-23).

### Statistical Analysis

Statistical analyses were performed using IBM® SPSS software version 25. Descriptive statistics were presented as frequency (percent), mean ± SD, or median (min-max). The χ^2^ and Exact tests were used

to compare the proportions in different categorical groups. Continuous variables were investigated with visual and analytical methods to determine the normal distribution and analyzed with the Student`s t-test. An overall type-1 error level was used to infer statistical significance.

## RESULTS

### Baseline Characteristics

Thirty-three patients (17 men, 16 women) with a mean age of 37.7 ± 11.4 years were included in the study. Within our cohort, the most predominant clinical phenotype was primary antibody deficiencies, representing 90.9%. Only two patients had a genetic diagnosis, and these patients were brothers diagnosed with Wiskott-Aldrich syndrome (WAS). A comprehensive breakdown of IEI diagnoses is presented in Figure 1. At diagnosis, the primary symptom prompting patients to seek medical attention was recurrent, prolonged, and severe infections, making up 93.9% of the cases. Among these, RTIs were most prevalent, with lower RTIs seen in 24 patients (72.7%) and upper RTIs (URTI) in 21 patients (63.6%). The two most common organ/ system comorbidities secondary to IEI in our patients were bronchiectasis in 16 (48.5%) patients and autoimmune disease in 13 (39.4%) patients. Detailed reasons for patients’ admissions at the time of diagnosis, and demographic, clinical, and laboratory characteristics are provided in Table 1. All patients underwent IGRT, with 31 receiving intravenous and two subcutaneous administration. Furthermore, 27 patients (81.8%) were on prophylactic antibiotics. 21 patients were prescribed trimethoprim- sulfamethoxazole, four patients were given azithromycin, one patient was on aciclovir, and one patient was on both valganciclovir and trimethoprim- sulfamethoxazole. Data on lymphocyte counts, peripheral blood lymphocyte subgroups, Ig levels at the time of diagnosis, and IgG levels during immunoglobulin therapy are presented in Table 1.

### Vaccination Against Pathogens Related to Respiratory Tract

The vaccination rate of patients against RTIs was 42.4%, 57.6%, and 78.8% for influenza, pneumococcus, and COVID-19, respectively (Figure 2a). Almost 80% (78.8%) of the patients were vaccinated against the SARS-CoV-2 pathogen, and 33.3% contracted a documented COVID-19 infection.

**Figure 1.** Classification of human inborn errors of immunity according to phenotype (7). Ig: Immunoglobulin.

**Table:** 

**Table 1.** Demographic and laboratory characteristics of inborn errors of immunity patients
Age € (years) 37.7 ± 11.4 Gender (Male), n (%) 17 (51.5%) Consanguineous marriages, n (%) 10 (30.3%) Smoking, n (%) 9 (27.3%) Diagnosis of IEI, n (%) Age of symptom onset€, years 18 ± 13.5 CVID 23 (69.7%) Age of diagnosis€, years 27 ± 13.7 Others 10 (30.3%) Diagnosis delay*, years 6 (0-33) Infections at the time of diagnosis, n (%) 31 (93.9%) Clinical features n (%) LRTI 8 (24.2%) Bronchiectasis 16 (48.5%) URTI 5 (15.1%) GIS involvement 8 (24.2%) LRTI+URTI 16 (48.4%) Atopy 7 (21.2%) UTI 2 (6%) Autoimmunity 13 (39.4%) Others 4 (12.1%) Hematological involvement 8 (24.2%) IGs at diagnosis, mg/dL Lymphocytes€, 109/L 1851 ± 836 IgG* (700-1600)x 445 (3-900) CD3€, % 76.6 ± 9.8 IgA* (70-400)x 6.7 (6-293) CD4, % 46.1 ± 17.1 IgM* (40-230)x 29 (4-775) CD8, % 39.8 ± 15.9 CD19€, % 8.4 ± 4.5 Trough IgG€, mg/dL 826 ± 239 CD16/CD56€, % 13.2 ± 8.1 CD3-HLADR€, % 5.6 ± 3.9
BMI: Body mass index, CVID: Common variable immunodeficiency, IEI: Inborn errors of immunity, IGs: Immunoglobulins, GIS: Gastrointestinal system, LRTI: Lower respiratory tract infection, URTI: Upper respiratory tract infection, UTI: Urinary tract infection, mg: Milligram; dL: Deciliter. x Reference ranges in our laboratory, continuous variables presented with *median (min-max) or €mean ± SD.

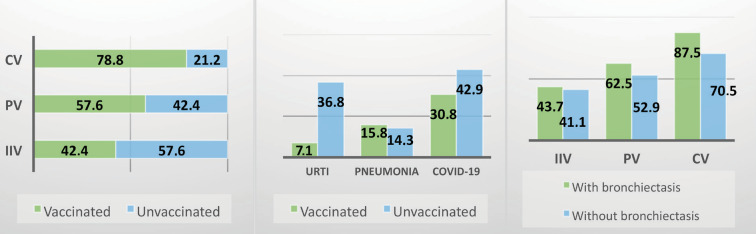

**Figure 2. A.** Vaccination rate (%) of inborn errors of immunity patients, **B.** The rates (%) of disease related to the specific agent among vaccinated and unvaccinated patients, **C.** The vaccination rates (%), stratified based on the presence or absence of bronchiectasis. IIV: Inactivated influenza vaccine, PV: Pneumococcal vaccine, CV: COVID-19 vaccine, URTI: Upper respiratory tract infections.

There was no statistically significant difference between those who received the COVID-19 vaccine and those who did not in terms of infection incidence or hospital visitations (n= 8, 30.8% vs. n= 3, 42.9%, p= 0.661). No patients reported side effects or allergic reactions attributed to the COVID-19 vaccine. Of the 33 patients, five (15.2%) developed pneumonia, and eight (24.2%) experienced URTI during the follow-up. The rates of RTIs among vaccinated and unvaccinated patients are shown in Figure 2b. In comparing the clinical phenotypes, demographic data, and laboratory results between patients who experienced respiratory infections and those who did not, we found no statistically significant differences. However, vaccination rates among patients with bronchiectasis were notably higher compared to those without bronchiectasis, with a particular emphasis on COVID-19 vaccination. The vaccination rates, stratified based on the presence or absence of bronchiectasis, are depicted in Figure 2c. The presence of bronchiectasis in our patients was not associated with a statistically significant difference in terms of contracting URTI and pneumonia (p= 1.000 and p= 0.656, respectively). However, strikingly, COVID-19 infections were observed less frequently in our patients with bronchiectasis (n= 2, 18.2% vs. n= 14, 63.6%, p= 0.014).

## DISCUSSION

In a cohort of 33 IEI patients receiving IGRT, we examined vaccination rates for respiratory pathogens: 42.4% for influenza, 57.6% for pneumococcus, and 78.8% for COVID-19. This study specifically focuses on patients with IEI who underwent IGRT, aiming to compare their vaccination status against RTI

pathogens and the disease rates between vaccinated and unvaccinated patients.

The purpose of vaccination is to induce a specific response against microorganisms and provide specific protection against them. According to data obtained from studies involving the general population, current influenza vaccines primarily stimulate humoral immunity but also activate cellular immunity. This dual stimulation is crucial, as cellular immunity plays an important role in clearing the virus and preventing disease-related complications (15,16). In our study, the most common phenotype among our patients with IEI was CVID (90.9%), which is the most prevalent clinically significant antibody deficiency disorder in adults (17). There are few studies evaluating the effectiveness of inactivated influenza vaccines (IIV) in CVID patients. Additionally, the number of patients included in these studies is very small. To our knowledge, there is no study evaluating the clinical endpoints of immunocompromised patients who received both IGRT and IIV. Although existing studies assessing humoral and cellular responses in patients with IEI provide limited data on vaccine effectiveness, available evidence suggests that vaccination does offer some benefits (18-21).

The seasonal IIV rate among our patients was 42.4%. An attempt was made to obtain information from hospital records regarding whether the patients had influenza during the season they were vaccinated. During the subsequent influenza season after vaccination, URTIs were observed in one vaccinated patient (7.1%) and seven non-vaccinated patients (36.8%). In both groups, patients did not require

hospitalization. Since microbiological and/or serological tests were not conducted on patients with a history of URTI, a comparison of vaccine protection against influenza was not feasible. Nevertheless, we believe that the fact that only one of our vaccinated patients had mildly symptomatic URTI, along with the data from the literature, indicates the potential protective role of IIV. Therefore, in line with recommendations, we advocate for seasonal IIV vaccination in IEI patients and their close contacts (2-5).

In CVID patients on IGRT, protective passive immunity is observed against pneumococcus and HIB, with variable protection against meningococcus (10,12,22). Additionally, variable responses to polysaccharide or conjugate vaccines are noted in this patient group (10- 12). For instance, a study with 23 CVID patients on IGRT showed that 23% responded to peptide or conjugate vaccines and 18% to polysaccharide vaccines (10). Another study in a similar cohort revealed that 65% had reduced yet protective responses to the meningococcal polysaccharide vaccines (11). This indicates significant variability in vaccine response among CVID patients on IGRT. While some individuals demonstrate favorable antibody responses to certain vaccines, notably peptide or conjugate vaccines, others may exhibit attenuated yet still protective responses to polysaccharide vaccines. Pneumonia developed in five of our 33 patients (15.2%) receiving IGRT therapy, and although specific pathogens could not be identified in our study, *Streptococcus pneumoniae* is typically a common causative agent (23). In the guidelines for vaccination for patients with IEI, pneumococcal vaccination is recommended for most primary antibody deficiencies and partial combined immunodeficiencies (e.g., WAS), even though its efficacy has not been clearly documented in conditions like X-linked agammaglobulinemia and CVID (4).

During the SARS-CoV-2 pandemic, initial immunoglobulin products had limited SARS-CoV-2 antibodies (13). Recent formulations include these antibodies, yet their protective efficacy is uncertain (13,24). Given the uncertainty surrounding the protective capacity of immunoglobulin therapy against this novel virus, ESID, and other immunology societies have issued a statement recommending COVID-19 vaccines for all individuals with primary immunodeficiencies, emphasizing the use of non- live vaccines. Studies suggest that CVID patients can

generate specific immune responses to SARS-CoV-2 (13).

Almost 80% (78.8%) of patients in our study were vaccinated against SARS-CoV-2 and 33.3% contracted COVID-19. In a retrospective study evaluating the vaccination rates among 681 healthcare workers who contracted COVID-19, it was reported that 53.3% of this cohort had been vaccinated (25). When compared to this study, healthcare workers, representing a group with a high risk of exposure to COVID-19, had notably lower vaccination rates than our patient cohort (78.8% vs. 50.5%). A study conducted in the United Kingdom indicated that vaccinated IEI patients had lower hospitalization and mortality rates than the unvaccinated (p< 0.0001 and p= 0.01, respectively) (26). However, our data did not reveal significant differences in infection or hospitalization rates post-vaccination, possibly due to limitations in sample size. When examining the impact of underlying bronchiectasis on COVID-19 infection, we observed a lower incidence among patients with bronchiectasis. This finding suggests that a higher rate of COVID-19 vaccination among patients with bronchiectasis might be a contributing factor (87.5% in those with bronchiectasis versus 70.5% in those without, Figure 2c). Pandemic-driven public advisories and media coverage significantly boosted COVID-19 vaccination uptake, particularly among bronchiectasis patients. This increase is likely due to heightened awareness of the risks associated with chronic lung conditions. Factors such as travel requirements and employer support also contributed to higher vaccination rates, especially when compared to other vaccines. Moreover, according to our retrospective data, no side effects, including allergic reactions, attributable to the COVID-19 vaccine were reported. This finding supports the safety of the aforementioned vaccine among a vulnerable population suffering from IEI.

Our study had several notable limitations. The retrospective nature of the study posed challenges, including a small patient sample and incomplete data on the pathogens responsible for pneumonia and URTI. These limitations hindered a comparative analysis of vaccine effectiveness between vaccinated and unvaccinated groups. Additionally, data gaps on the type of pneumococcal vaccine received and the inability to measure post-vaccination IgG levels further constrained our findings. However, the strength of our study is that it draws attention to the importance

and safety of inactivated vaccinations in this unique patient population and incorporates current literature recommendations. While the limited sample size constrains our ability to draw definitive conclusions, the data underscores the pressing need for sustained vaccination efforts for this susceptible group to shield them from preventable infections. Increasing seasonal inactive influenza vaccination rates among the IEI population to reach 80%, similar to COVID-19 vaccination rates, is achievable through public advisories, which may lead to reduced mortality and morbidity.

## CONCLUSION

In conclusion, our analysis of IEI patients undergoing IGRT revealed high COVID-19 vaccination rates, and there is evidence suggesting that vaccination reduces symptoms of respiratory infections. This study also demonstrates the potential to achieve higher vaccination rates for recommended vaccinations through social and governmental influence. It is important to note that the management of vaccination in IEI patients is highly individualized, and recommendations may vary based on the specific type and severity of the immune deficiency. Further studies are warranted to assess the safety and effectiveness of vaccines in these special patient groups.

**Ethical Committee Approval:** This study was approved by the Ankara University Human Researchs Ethics Committee (Decision no: İ04-229-23, Date: 18.04.2023).

### CONFLICT of INTEREST

The authors declare that they have no conflict of interest.

## AUTHORSHIP CONTRIBUTIONS

Concept/Design: All of authors Analysis/Interpretation: All of authors Data acqusition: All of authors Writing: MSBD, SA, RY

Clinical Revision: MSBD, SA Final Approval: MSBD, SA

## REFERENCES

1. *
  Pieniawska-Smiech K, Pasternak G, Lewandowicz- Uszynska A, et al. Diagnostic Challenges in Patients with Inborn Errors of Immunity with Different Manifestations of Immune Dysregulation. J Clin Med 2022; 11: 20220720. https://doi.org/10.3390/jcm11144220*
2. *
  Sobh A, Bonilla FA. Vaccination in primary immunodefi- ciency disorders. J Allergy Clin Immunol Pract 2016; 4(6): 1066-75. https://doi.org/10.1016/j.jaip.2016.09.012*
3. *
  Bonilla FA. Update: Vaccines in primary immunodeficien- cy. J Allergy Clin Immunol 2018; 141(2): 474-81. https:// doi.org/10.1016/j.jaci.2017.12.980*
4. *
  Shearer WT, Fleisher TA, Buckley RH, Ballas Z, Ballow M, Blaese RM; Medical Advisory Committee of the Immune Deficiency Foundation, et al. Recommendations for live viral and bacterial vaccines in immunodeficient patients and their close contacts. J Allergy Clin Immunol 2014; 133(4):961-6.https://doi.org/10.1016/j.jaci.2013.11.043*
5. *
  Rubin LG, Levin MJ, Ljungman P, Davies EG, Avery R, Tomblyn M, et al. Infectious diseases society of America. 2013 IDSA clinical practice guideline for vaccination of the immunocompromised host. Clin Infect Dis 2014; 58(3): 309-18. https://doi.org/10.1093/cid/cit816*
6. *
  National Center for Immunization and Respiratory Diseases. General recommendations on immunization - recommendations of the Advisory Committee on Immunization Practices (ACIP). MMWR Recomm Rep 2011; 60(2): 1-64.
  *
7. *
  Tangye SG, Al-Herz W, Bousfiha A, Cunningham-Rundles C, Franco JL, Holland SM, et al. Human inborn errors of immunity: 2022 update on the classification from the International Union of Immunological Societies Expert Committee. J Clin Immunol 2022; 42(7): 1473-507. https://doi.org/10.1007/s10875-022-01289-3*
8. *
  Guevara-Hoyer K, Saz-Leal P, Diez-Rivero CM, Ochoa- Grullón J, Fernández-Arquero M, Pérez de Diego R, et al. Trained immunity based-vaccines as a prophylactic strate- gy in common variable immunodeficiency. A proof of concept study. Biomedicines 2020; 8(7): 203. https://doi. org/10.3390/biomedicines8070203*
9. *
  Mieves JF, Wittke K, Freitag H, Volk HD, Scheibenbogen C, Hanitsch LG. Influenza vaccination in patients with com- mon variable immunodeficiency (CVID). Curr Allergy Asthma Rep 2017; 17(11): 78. https://doi.org/10.1007/ s11882-017-0749-3*
10. *
  Goldacker S, Draeger R, Warnatz K, Huzly D, Salzer U, Thiel J, et al. Active vaccination in patients with common variable immunodeficiency (CVID). Clinical Immunology 2007; 124(3): 294-303. https://doi.org/10.1016/j. clim.2007.04.011*
11. *
  Rezaei N, Siadat SD, Aghamohammadi A, Moin M, Pourpak Z, Norouzian D, et al. Serum bactericidal anti- body response 1 year after meningococcal polysaccharide vaccination of patients with common variable immunode- ficiency. Clin Vac Immun 2010; 17(4): 524-8. https://doi. org/10.1128/CVI.00389-09*
12. *
  Adam E, Church JA. Antibody levels to Bordetella pertussis and Neisseria meningitidis in immunodeficient patients receiving immunoglobulin replacement therapy. J Clin Immunol 2015; 35: 213-7. https://doi.org/10.1007/ s10875-015-0131-y*
13. *
  ESID. COVID-19-statement-March-2022. Available from: https://esid.org/COVID-19/ESID-COVID-19-Statement- March-2022.
  *
14. *
  Seidel MG, Kindle G, Gathmann B, Quinti I, Buckland M, van Montfrans J, et al. The European Society for Immunodeficiencies (ESID) registry working definitions for the clinical diagnosis of inborn errors of immunity. J Allergy Clin Immunol Pract 2019; 7(6): 1763-70. https:// doi.org/10.1016/j.jaip.2019.02.004*
15. *
  Cox RJ, Brokstad KA, Ogra P. Influenza virus: Immunity and vaccination strategies. Comparison of the immune response to inactivated and live, attenuated influenza vaccines. Scand J Immunol 2004; 59(1): 1-15. https://doi. org/10.1111/j.0300-9475.2004.01382.x*
16. *
  Rappuoli R. Bridging the knowledge gaps in vaccine design. Nat Biotechnol 2007; 25(12): 1361-6. https://doi. org/10.1038/nbt1207-1361*
17. *
  Gathmann B, Grimbacher B, Beauté J, Dudoit Y, Mahlaoui N, Fischer A, et al; ESID Registry Working Party. The European internet-based patient and research database for primary immunodeficiencies: Results 2006-2008. Clin Exp Immunol 2009; 157 Suppl 1(Suppl 1): 3-11. https:// doi.org/10.1111/j.1365-2249.2009.03954.x*
18. *
  Committee for Proprietary Medicinical Products CPMP. Note for guidance on harmonisation of requirements for influenza vaccines. European agency for the evaluation of medicinal products (EMEA), London. 1997. http://www. ema. europa. eu/ docs/ en_GB/ document_library/ Scientific_guideline/2009/09/WC500003945.pdf.
  *
19. *
  van Assen S, Holvast A, Telgt DS, Benne CA, de Haan A, Westra J, et al. Patients with humoral primary immunode- ficiency do not develop protective anti-influenza antibody titers after vaccination with trivalent subunit influenza vaccine. Clin Immunol (Orlando, Fla) 2010; 136(2): 228-
  *

*
35. https://doi.org/10.1016/j.clim.2010.03.430*

20. *
  Hanitsch LG, Löbel M, Mieves JF, Bauer S, Babel N, Schweiger B, et al. Cellular and humoral influenza-specific immune response upon vaccination in patients with com- mon variable immunodeficiency and unclassified anti- body deficiency. Vaccine 2016; 34(21): 2417-23. https:// doi.org/10.1016/j.vaccine.2016.03.091*
21. *
  Pedersen G, Halstensen A, Sjursen H, Naess A, Kristoffersen EK, Cox RJ. Pandemic influenza vaccination elicits influen- za-specific CD4+ Th1-cell responses in hypogammaglob- ulinaemic patients: Four case reports. Scand J Immunol 2011; 74(2): 210-8. https://doi.org/10.1111/j.1365-*

*3083.2011.02561.x*

22. *
  Chua I, Lagos M, Charalambous BM, Workman S, Chee R, Grimbacher B. Pathogen-specific IgG antibody levels in immunodeficient patients receiving immunoglobulin replacement do not provide additional benefit to thera- peutic management over total serum IgG. J Allergy Clin Immunol 2011; 127: 1410-1. https://doi.org/10.1016/j. jaci.2011.01.035*
23. *
  Hong R, Agrawal S, Gollapudi S, Gupta S. Impaired pneu- movax-23-induced monocyte-derived cytokine produc- tion in patients with common variable immunodeficiency. J Clin Immun 2010; 30(3): 435-41. https://doi. org/10.1007/s10875-010-9371-z*
24. *
  Volk A, Covini-Souris C, Kuehnel D, De Mey C, Römisch J, Schmidt T. SARS-Cov-2 neutralization in convalescent plasma and commercial lots of plasma-derived immuno- globulin. Biodrugs 2022; 36(1): 41-53. https://doi. org/10.1007/s40259-021-00511-9*
25. *
  Kazcı S, Mirza A, Çöl M, Baysan C, Soysal Ç, Yılmaz E, et al. Bir eğitim ve araştırma hastanesinde COVID-19 geçiren sağlık çalışanlarında aşılanma durumu ve klinik özellikler. ESTÜDAM Halk Sağlığı Derg 2023; 8(2): 206-19. https:// doi.org/10.35232/estudamhsd.1274240*
26. *
  Shields AM, Tadros S, Al-Hakim A, Nell JM, Lin MMN, Chan M, et al. Impact of vaccination on hospitalization and mortality from COVID-19 in patients with primary and secondary immunodeficiency: The United Kingdom experience. Front Immunol 2022; 13: 984376. https://doi. org/10.3389/fimmu.2022.984376*

